# Proprietary Milk Protein Concentrate Reduces Joint Discomfort While Improving Exercise Performance in Non-Osteoarthritic Individuals

**DOI:** 10.3390/nu11020283

**Published:** 2019-01-28

**Authors:** Tim N. Ziegenfuss, Chad M. Kerksick, A. William Kedia, Jennifer Sandrock, Betsy Raub, Hector L. Lopez

**Affiliations:** 1 The Center for Applied Health Sciences, 4302 Allen Road, Suite 120, Stow, OH 44224, USA; Awkedia@sbcglobal.net (A.W.K.); jh@appliedhealthsciences.org (J.S.); br@appliedhealthsciences.org (B.R.); hl@appliedhealthsciences.org (H.L.L.); 2 Exercise and Performance Nutrition Laboratory, School of Health Sciences, Lindenwood University, 209 S. Kingshighway, St. Charles, MO 63301, USA; ckerksick@lindenwood.edu

**Keywords:** milk, protein, immunity, inflammation, concentrate, joint health, osteoarthritis

## Abstract

Milk and dairy products are known to contain various bioactives with potential anti-inflammatory and immune modulating effects. Previous research has indicated that milk produced from hyperimmunized cows provided meaningful health benefits to individuals suffering from varying degrees of osteoarthritis and rheumatoid arthritis. **PURPOSE:** To examine the impact of a proprietary milk protein concentrate on joint discomfort and physical function, exercise performance, quality of life and various measures of affect. **METHODS:** Non-osteoarthritic men (42.5 ± 8.9 years, 176.7 ± 6.7 cm, 89.9 ± 11.5 kg, 28.8 ± 3.5 kg/m^2^, *n* = 30) and women (46.4 ± 9.6 years, 163.1 ± 8.2 cm, 72.2 ± 13.1 kg, 27.2 ± 5.3 kg/m^2^, *n* = 28) with mild to moderate knee pain during physical activity were randomized in a double-blind, placebo-controlled fashion to consume daily either a placebo (PLA) or proprietary milk protein concentrate (MP) for a period of 8 weeks. Participants completed a functional capacity test pre and post-supplementation and completed visual analog scales (VAS), a 6-min walking test, WOMAC and profile of mood states (POMS) to assess changes in joint health, discomfort, physical function, exercise performance and affect. Mixed factorial ANOVA was used for all statistical analysis and significance was set *a priori* at *p* ≤ 0.05. **RESULTS:** Distance covered in the 6-min walking significantly improved 9% in MP versus 2% in PLA (mean difference: 110 ± 43 m, *p* = 0.012) in addition to 11 WOMAC components and 5 VAS reflective of MP improving joint health, discomfort and joint stability (all *p* < 0.05 vs. PLA). Additionally, MP also improved overall perceptions of neck and back health compared to PLA. Serum and whole blood indicators of clinical safety remained within normal ranges throughout the study. **CONCLUSIONS:** In comparison to placebo, daily doses of proprietary milk protein concentrate yielded improvements in several components of the WOMAC, multiple visual analog scales indicative of joint health and stability, discomfort and pain, as well as significant improvements in distance covered during a 6-min walking test. Supplementation was well tolerated with no significant changes in whole-blood or serum markers of clinical safety.

## 1. Introduction

According to the results of a National Health Interview Survey released in 2015, approximately 91 million people have some form of arthritis [1]. Of the different forms of arthritis, osteoarthritis is the most common joint disorder in the United States with approximately 10% of men and 13% of women over 60 years of age having symptomatic knee osteoarthritis [2,3]. By the year 2030, it is estimated that 20% of U.S. Americans (approximately 70 million people) will reach the age of 65, the point of increased risk of osteoarthritis. Key symptoms associated with osteoarthritis include the onset of pain, development of various disabilities such as increased difficulty with climbing stairs, walking and other activities of daily living [4,5]. As such, the combination of advancing age in the population and the established prevalence of pain-related symptoms and lost productivity results in the need for primary and secondary means to alleviate pain and improve function [6]. In this respect, pharmacological approaches and non-steroidal anti-inflammatory drugs (NSAIDS) including acetaminophen, ibuprofen and naproxen are commonly considered [7]. In addition to prescription and non-prescription medications, several nutraceuticals are commonly considered for their ability to mitigate pain (discomfort) and inflammation including glucosamine, chondroitin, hyaluronic acid, calcium, omega-3 fatty acids, olive oil and curcumin [8]. Beyond these approaches, several studies have highlighted milk from hyper-immunized cows, for their ability to bolster immunity and promote anti-inflammation [9,10,11,12]. Additionally, both human and bovine milk are known to contain many bioactive factors [13] while hydrolyzing casein and whey (proteins found in milk) yields several bioactive peptides with a diverse array of biological functions/effects, including opioid agonists and antagonists, antimicrobial, anti-inflammatory, immune modulations and nutrient transport systems [14].

Previous research has shown that the ingestion of skim milk powder from hyperimmunized cows can reduce cholesterol [9] and inflammation [10,11]. While initial outcomes were positive (improved ratings of QOL, energy, general well-being and sleep quality in over 60% of the 3500 people surveyed), the open-label, cross-sectional nature of the original report [15], impracticality of dosing and inconvenience associated with poor mixing and palatability stymied additional investigation. To address these shortcomings, a proprietary (lactose-free) milk protein concentrate was developed from milk produced in hyperimmunized cows retaining much of the valuable high-molecular weight immunoglobulins and low-molecular weight bioactives while removing much of the carbohydrate, salt and fat resulting in its ability to be concentrated and encapsulated. To examine its effects, Colker et al. [16] had 31 adults with physician diagnosed osteoarthritis in their knees supplement in a randomized, double-blind fashion with either a placebo or concentrated milk protein. Before and after supplementation, participants completed visual analog scales for pain, physical function and quality of life while also completing the Western Ontario and McMaster Universities Osteoarthritis Index (WOMAC). A significant treatment effect was observed in the WOMAC, knee pain, sport function and quality of life for those participants who consumed the concentrated milk protein while only improvements in sport function and quality of life were found in the placebo group. Additional research by Zenk et al. [17] investigated the impact of ingesting a concentrated milk protein from hyperimmunized cows in 35 men and women (range: 34–86 years) diagnosed with osteoarthritis who were supplemented in a randomized, double-blind fashion to ingest either concentrated milk protein (two daily doses of 2000 mg), glucosamine sulfate (three daily doses of 500 mg) or a placebo for a six-week period. In this study, osteoarthritis symptoms were again assessed using the WOMAC. As seen with the Colker study, those individuals who supplemented with a concentrated milk protein from hyperimmunized cows reported a significant reduction in all four scores of the WOMAC (i.e., stiffness, discomfort, physical function and total score). Glucosamine supplemented participants experienced significant reductions in two of the four scores (stiffness and total WOMAC) while no changes were reported in the placebo group.

Research to date on ingestion of concentrated milk proteins from milk produced by hyperimmunized cows has been limited to individuals with osteoarthritis. In addition, while the Zenk [17] and Colker [16] findings are valuable due to the randomized, double-blind, placebo controlled study approach they employed, both studies recruited relatively small sample sizes (*n* = 10–15 per group) and failed to examine if supplementation can improve performance of a physical task and if pain or discomfort associated with completing that task was reduced. Furthermore, beyond markers of clinical safety, neither study reported on any biomarkers that might help to offer any insight into potential mechanism(s) of action. Therefore, the purpose of this study was to examine the impact of ingesting a concentrated milk protein derived from the milk produced by hyperimmunized cows on alleviating pain (discomfort) and function with and without an external physical stimulus in non-osteoarthritic participants who reported having mild to moderate functional knee pain during/after physical activity. We also sought to examine changes in physical performance and a biomarker of cartilage breakdown.

## 2. Methods

### 2.1. Overview of Study Design

The study was a randomized, double-blind, placebo-controlled investigation using two parallel supplementation groups that spanned eight weeks. Each participant completed four study visits. The first visit was for screening purposes and consisted of signing an IRB-approved consent form, completing a medical history, recording their diet information, completing the functional capacity test and visual analog scales and having routine blood work (CMP, CBC, lipid panel) completed. Prior to the screening visit, participants were confirmed to have stopped, for the previous four weeks, the use of over-the-counter medicines for pain or inflammation, including non-steroidal anti-inflammatory drugs (NSAIDS), acetaminophen and full-dose aspirin (325 mg). At regular four-week intervals, study participants completed three additional study visits (0, 4 and 8 weeks) and were assessed for dietary habits, physical activity (Framingham Physical Activity Index), adverse events, profile of mood states (POMS), joint pain and health (WOMAC) and various visual analog scales to assess fatigue/energy, mood, quality of training and motivation to exercise. In addition, participants completed a functional capacity test as a part of visits 3 (week 4) and 4 (week 8). To evaluate clinical safety, participants had hemodynamic, complete blood counts, comprehensive metabolic and lipid panels completed during visits 1 (week 0) and 4 (week 8). Prior to all study visits, participants were asked to replicate their previous 24-hr dietary intake, avoid alcohol for 24 h, abstain from exercise for 48 h and fast for ten hours. To control for diurnal variations, all study visits were completed in the morning (700–1200) with all follow-up visits being scheduled at a similar time as initial visits. Upon completion of visits 2 and 3, participants were provided with study product. Table 1 provides a general layout of all testing. This clinical trial did not evaluate a drug, biological product or medical device and consequently was not required to be registered at Clinicaltrials.gov prior to its initiation.

### 2.2. Study Participants

Male (42.5 ± 8.9 years, 176.7 ± 6.7 cm, 89.9 ± 11.5 kg, 28.8 ± 3.5 kg/m^2^, *n* = 30) and female (46.4 ± 9.6 years, 163.1 ± 8.2 cm, 72.2 ± 13.1 kg, 27.2 ± 5.3 kg/m^2^, *n* = 28) study participants between the ages of 35–70 years were recruited from the local community (Table 2). Prior to any data collection, all subjects gave their informed consent for inclusion before they were enrolled into the study. The study was conducted in accordance with the Declaration of Helsinki and the protocol was approved by the Ethics Committee of Integreview (Integreview, Austin, TX; protocol # SMB001-2017, approval date: December 13, 2017). Study participants were pre-screened using health history questionnaires, had vital signs assessed and a physical exam was completed by a physician. Eligible study participants were those individuals who were free of disease, including osteoarthritis, between the ages of 35–70 years, non-smokers, meeting minimum physical activity recommendations and had a body mass index between 27–45 kg/m^2^ at screening. All participants were required to self-report no knee joint discomfort at rest and ‘mild-to-moderate or higher’ (defined as a rating of 30/100 or higher on a 100-mm visual analog scale for knee discomfort) with activity or exercise over the last three weeks. Females were required to not be pregnant or currently lactating and agree to use an effective contraceptive method during the study. Changes in menstrual cycle were viewed to be minimal due to each study visit being eight weeks apart. Each visit was scheduled at similar times of the day to further help control for any diurnal variations. Participants were excluded if they: were already diagnosed with having some form of inflammatory disorder or osteoarthritis, currently taking some form of anti-inflammatory nutritional supplement, had received any form of intraarticular injection (glucocorticoid, hyaluronic acid, bone marrow, platelet rich plasma, etc.) within six months of enrolling in study, reported daily use of a non-steroidal anti-inflammatory drug, were determined to have anemia or another form of nutritional deficiency, psychiatric illness, GI disorder, allergic reactions to bovine milk or other food allergies or intolerances or abnormal vital signs (systolic blood pressure: <90 or >150 mm Hg, diastolic blood pressure: <50 or >100 mm Hg, heart rate: <50 or >110 beats per minute).

### 2.3. Demographics

Standing height was determined using a wall-mounted stadiometer with each study participant in socks with their heels together. Body weight was measured using a Seca 767™ Medical Scale (Hamburg, Deutschland). Resting heart rate and blood pressure was measured in duplicate using an automated blood pressure cuff (Omron HEM-780).

### 2.4. Venous Blood Collection

Whole blood and serum samples were collected using standard phlebotomy techniques during all study visits after participants observed a ten hour fast. All collected samples were processed immediately for analysis at a central clinical chemistry laboratory (LabCorp) while a portion of remaining sample was stored at −80 °C for any remaining analyses. Whole blood samples were collected into K_2_-EDTA treated Vacutainer tubes and upon collection were slowly inverted ten consecutive times prior to immediate refrigeration. Serum samples were collected in non-treated tubes and allowed to clot for 30 min at room temperature prior to being centrifuged (Horizon mini E Centrifuge, Drucker Diagnostics, Port Matilda, PA) for 15 min at 3200 rpm (1500× *g*). Serum was extracted from all samples and aliquots were pipetted into cryovial storage tubes. All blood samples were analyzed for clinical chemistry analysis (plasma glucose, blood urea nitrogen (BUN), creatinine, aspartate aminotransaminase (AST), alanine aminotransaminase (ALT), creatine kinase, lactate dehydrogenase, total bilirubin, alkaline phosphatase (ALP), triacylglycerol (TG), total cholesterol [TC], LDL-C, HDL-C, uric acid, sodium, potassium, total protein, albumin, globulin, iron, complete blood cells and platelet count). All samples from the same day were batch analyzed with test-retest reliabilities commonly reported using internal quality control data from clinical laboratories and associated automated analyzers within a range of 3–5% [18]. Serum levels of cartilage oligomeric matrix protein (COMP) were analyzed in duplicate using standard ELISA techniques. The test-retest reliability coefficient for this assay was <10%.

### 2.5. Functional Capacity

Functional capacity was assessed using a standardized protocol as part of screening and again at week 4 and week 8. After completing a basic warm-up consisting of ten minutes of whole-body stretches and callisthenic movements, study participants performed three sets of 10–12 repetitions of leg extension exercise. These repetitions were completed as an attempt to pre-fatigue the involved musculature. The load was assigned to yield an RPE level of at least an 8 (out of 10) during the completion of the last two repetitions of each set. Load was adjusted after the first set of repetitions if a minimum RPE of 7 (out of 10) was not reported. Two minutes of rest were provided between each set. Upon completing the third set, study participants were required to complete a 6-min walk test at a self-selected maximal walking velocity. Prior to commencing the test, study participants were informed of the course layout and instructed to walk as far and as fast as they could for the six-minute duration of the test.

### 2.6. Visual Analog Scales and Questionnaires to Assess Affect and Joint Health

Visual analog scales (VAS) were completed by study participants at baseline, week 4 and week 8. All VAS were constructed similarly with a 100-mm line anchored by “Lowest Possible” and “Highest Possible” to assess subjective ratings of joint discomfort, knee mobility, knee stability, low-back health, neck mobility and whole-body joint health. The validity and reliability of VAS to assess fatigue and energy have been previously established [19]. At each study visit, the profile of mood states (POMS) was administered. The POMS consist of a series of five item questions (0—Not At All, 1—A little, 2—Moderately, 3—Quite a Lot, 4—Extremely) associated with negative (tension, depression, fatigue, confusion and anger) and positive (vigor, esteem) feelings to determine a total score. Additionally, participants were administered the Western Ontario and McMaster Universities Osteoarthritis Index (WOMAC). Subscale totals for discomfort, stiffness, physical function as well as a total score were computed.

### 2.7. Dietary Intake and Physical Activity

Participants were asked to maintain their current energy and macronutrient intake throughout the entire study protocol. During the initial screening visit, participants were asked to complete a 24-h dietary recall to assess general dietary habits, food restrictions, diet composition and quantity of intake. Additionally and as the study progressed, three-day dietary records were completed prior to visits two (baseline), three (week 4) and four (week 8) to track dietary compliance. Dietary records were analyzed for average daily energy and macronutrient intake by trained study investigators and NutriBase IX (Clinical Edition) software (CyberSoft, Inc. Phoenix, AZ, USA). Copies of food records were made and provided to each study participant to allow them to standardize their dietary and fluid intake 24 h prior to each study visit. At each study visit, a research team member asked each participant a series of questions using the Framingham Physical Activity Index regarding sleep, occupational and non-occupational activities to assess physical activity status [20,21].

### 2.8. Supplementation

In a randomized, double-blind fashion, all study participants were assigned to orally ingest, on a daily basis, either a placebo (microcrystalline cellulose) or 4000 milligrams of a proprietary milk protein concentrate from hyperimmunized cows. Both products were provided in opaque “00” sized capsules. Subjects took one dose (5 capsules totaling 2000 mg) in the morning and one dose (5 capsules totaling 2000 mg) in the evening with 12 fluid ounces of cold tap water. Supplement purity and potency was confirmed by a third party (independent) laboratory. All randomization was completed using an online randomization program. (www.randomizer.org).

### 2.9. Adverse Events

During weekly phone calls, the frequency and intensity of local and systemic non-serious and serious adverse events (AEs) were recorded by study team members. All reported events were coded using the Medical Dictionary for Regulatory Activities (MedDRA) while the intensity of recorded adverse events were graded using standardized criteria.

### 2.10. Statistical Analysis

All data were entered into Microsoft Excel (Seattle, WA, USA) software and analyzed using IBM SPSS 23 (Armonk, NY, USA). *A priori* power analysis and sample size determination was based on previous studies using the WOMAC [16,17]. For these calculations, an estimated mean difference of 4.5 for total WOMAC score was assumed to be a meaningful change in the MP group in comparison to the changes observed in the PLA group. With an alpha level of 0.05 and power set at 0.80, it was determined a sample size of 30 participants (SD = 10) were needed per group. Normality assumptions were checked on all variables using a one-sample Shapiro-Wilk test. Many variables assessed using VAS, POMS and WOMAC were found to be non-normally distributed. In these instances, findings were assessed first using Mann-Whitney U and when significant between-group differences were realized, Wilcoxon signed rank tests were completed to evaluate differences between time points. Due to the known robustness of the ANOVA and to aid in understanding identified changes, all non-normally distributed data was transformed using log_10_ and was subsequently presented and analyzed using parametric approaches. For example, independent t-tests were used to assess baseline differences. Data were initially analyzed using a 3-way mixed factorial ANOVA (gender x group × time) to assess gender specific responses in each group across time, however, no gender-specific outcomes were noted. While some key variables approached significance, none reached the *a priori* statistical threshold of 0.05. Consequently, all remaining data were analyzed using a 2 × 2 mixed factorial ANOVA (group (placebo vs. MP) × time (0 vs. 8 weeks)) with repeated measures on time to determine the presence of any main (time or group) and interaction (group × time) effects. No midpoint (4-week) analysis was computed due to an *a priori* determination to assess changes in dependent variables after eight weeks of supplementation. When the sphericity assumption was not met, the Huynh-Feldt correction was applied. Group × time interaction effects were decomposed using independent samples t-test using the calculated delta (Week 8–Week 0). Mean difference of the change scores and 95% confidence intervals were calculated on the difference between groups using delta values. Within-group effects were compared using paired samples t-test. Outliers were checked via visual inspection of studentized calculations on the residuals (threshold value of ± 3) of each dependent variable. Effects were considered significant at *P* ≤ 0.05 and trends were declared at 0.05 ≤ *P* ≤ 0.10. Effect sizes (eta squared) are also reported, using 0.01, 0.06, and 0.14 to represent small, medium and large effects, respectively. 

## 3. Results

**Figure 1:**
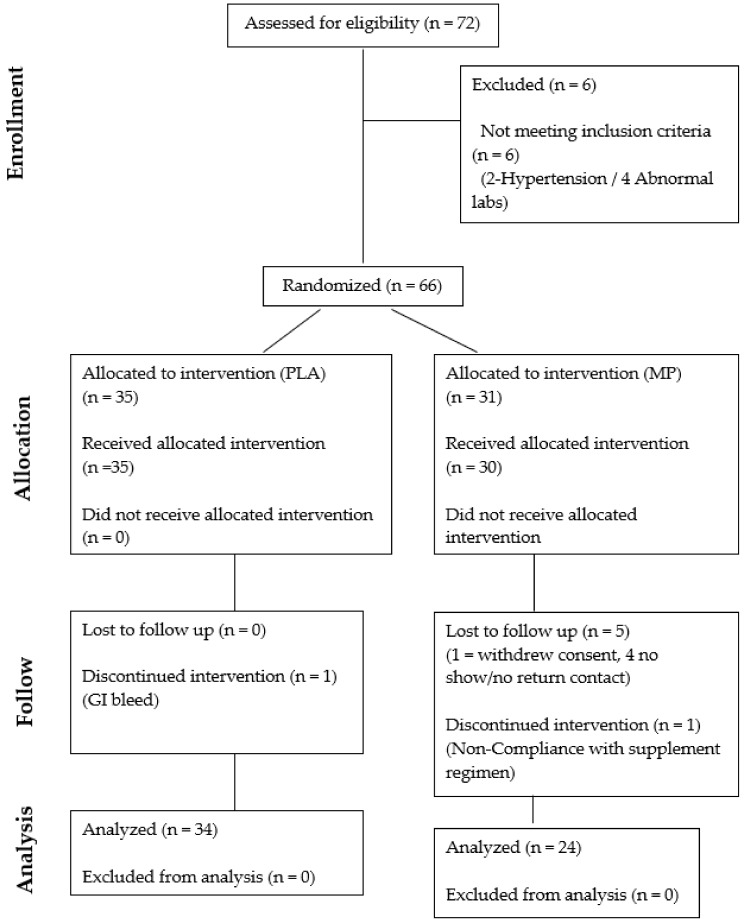
Consolidated Standards of Reporting Trials (CONSORT) diagram.

No baseline differences (p > 0.05) were noted between groups or between gender (sex) for any of the demographic variables (age, height, body mass, body mass index, blood pressure (systolic & diastolic), resting heart rate, 6-min walk test performance, WOMAC total stiffness, WOMAC physical function and WOMAC total score) (Table 2). One participant assigned to the placebo group experienced a gastrointestinal hemorrhage while enrolled in the study protocol. No other adverse events were reported for any other participants enrolled in the study (see Figure 1).

### 3.1. Dietary and Physical Activity Data

No significant main effect for time or group × time interaction effects were identified for any of the dietary data. Caloric intake was determined to be (PLA: 1,728 ± 502, MP: 1578 ± 413 kcals/day) and (PLA: 1819 ± 645, MP: 1581 ± 519 kcals/day) for visits week 0 and week 8, respectively. The main effect for time (*p* = 0.56) and the group × time interaction effect (*p* = 0.46) were not statistically significant. Carbohydrate intake was determined to be (PLA: 195 ± 75, MP: 160 ± 54 g/day) and (PLA: 199 ± 89, MP: 163 ± 56 g/day) for week 0 and week 8, respectively. The main effect for time (*p* = 0.38) and the group × time interaction effect (*p* = 0.99) were not statistically significant. Fat intake was determined to be (PLA: 68 ± 21, MP: 69 ± 26 g/day) and (PLA: 73 ± 46, MP: 65 ± 26 g/day) for week 0 and week 8, respectively. The main effect for time (*p* = 0.59) and the group × time interaction effect (*p* = 0.46) were not statistically significant. Protein intake was determined to be (PLA: 87 ± 28, MP: 80 ± 25 g/day) and (PLA: 91 ± 37, MP: 77 ± 25 g/day) for week 0 and week 8, respectively. The main effect for time (*p* = 0.27) and the group × time interaction effect (*p* = 0.44) were not statistically significant. Additionally, when macronutrient intake was expressed as a percentage of total calories consumed, no significant differences were noted for all macronutrients between groups across the duration of the study. Framingham physical activity index values were assessed at baseline (PLA = 38.6 ± 8.9 vs. MP = 37.7 ± 9.2, *p* = 0.71) and after completion (PLA: 38.9 ± 9.7 vs. MP: 38.0 ± 10.0, *p* = 0.75) of the study protocol. Additionally, no significant group × time interaction effect was found (*p* = 0.97). Intraclass correlation coefficient of the PLA group across all measurements timepoints were determined for total calorie (95% CI: 0.713–0.869), carbohydrate (95% CI: 0.786–0.906), fat (95% CI: 0.327–0.629) and protein (95% CI: 0.627–0.823) intake as well as Framingham Physical Activity Index (95% CI: 0.914–0.964).

### 3.2. WOMAC Data

**Figure 2:**
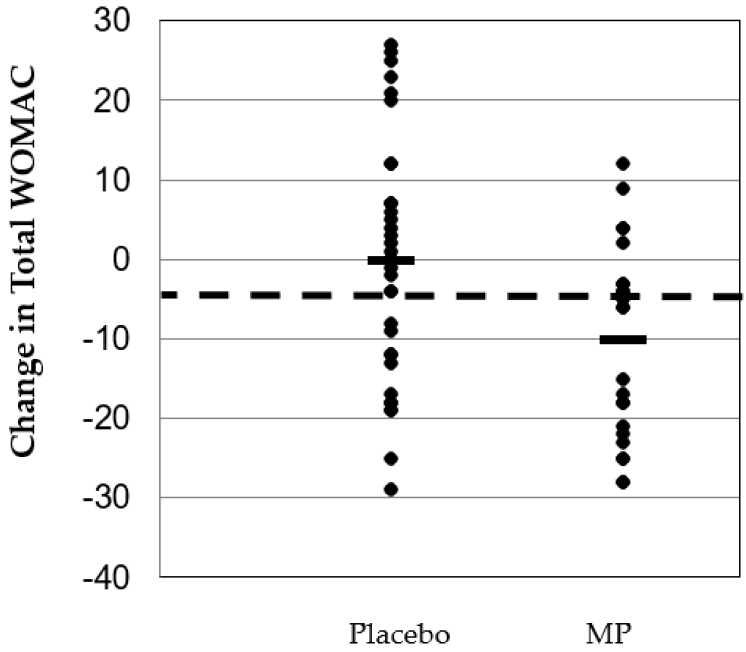
Changes in total WOMAC score after eight weeks of supplementation. The placebo group (*n* = 34) reported an average decrease in total WOMAC score of −0.26 points. Of the 34 scores in the PLA group, 12 (35.2%) scores experienced a reduction in their total WOMAC score more than the entire study average. The proprietary milk protein concentrate group (*n* = 24) reported an average decrease in total WOMAC score of 10.21 points. Of the 24 scores in the proprietary milk protein concentrate group, 15 (62.5%) scores decreased their total WOMAC score more than the entire study average. Dotted line = average change in total WOMAC for entire study cohort. Black horizontal bars = average change in total WOMAC for each study group.

Eleven WOMAC variables exhibited significant group × time interaction effects that favored the MP group: Physical Function – *Descending Stairs* (*p* = 0.02; 95% CI: 0.09, 1.17), Physical Function—*Standing* (*p* = 0.03; 95% CI: 0.05, 0.76), Physical Function—*Getting in Car* (*p* = 0.002; 95% CI: 0.25, 1.04), Physical Function—*Shopping* (*p* = 0.004; 95% CI: 0.20, 0.96), Physical Function—*Putting on Socks* (*p* = 0.002; 95% CI: 0.25, 1.04), Physical Function—*Removing Socks* (*p* = 0.002; 95% CI: 0.23, 1.00), Physical Function—*Moving in Bed* (*p* = 0.002, 95% CI: 0.21, 0.91), Physical Function—*Getting In and Out of Bed* (*p* = 0.005, 95% CI: 0.15, 0.78), Physical Function, *Getting On and Off the Toilet* (*p* = 0.011, 95% CI: 0.11, 0.80), Physical Function Total Score (*p* = 0.004, 95% CI: 2.8, 13.3) and WOMAC Total Score (*p* = 0.01, 95% CI: 2.5, 17.4). Percent improvement was 35% in overall joint health (Total WOMAC score) with MP compared to no change in PLA (~1% decrease). Physical function (i.e., mobility) improved 39% with MP compared to a 1% improvement in PLA. Other markers of physical function (i.e., shopping, getting in and out of bath, putting on socks, moving in bed, etc.) in the MP group experienced improvements of 48–60% compared to 3–17% improvements in PLA. In addition, five additional variables approached the statistical threshold for a statistically significant group × time interaction effect (*p* = 0.05–0.10). These variables included: Physical Function – *At Night While in Bed* (*p* = 0.054, 95% CI: −0.007, 0.79), Physical Function—*Ascending Stairs* (*p* = 0.062, 95% CI: (−0.03, 1.11), Physical Function—*Rising to Sitting* (*p* = 0.08, 95% CI: −0.06, 0.96), Physical Function—*Walking on Flat Surface* (*p* = 0.09, 95% CI: −0.06, 0.86) and Physical Function—*Sitting* (*p* = 0.074, 95% CI: −0.03, 0.71). All WOMAC data can be found in Table 3. Changes in total WOMAC scores are shown in Figure 2.

### 3.3. Visual Analog Scales

Five variables exhibited a statistically significant group × time interaction favoring the MP group: overall knee stability (*p* = 0.001), low back health (*p* = 0.002), neck health (*p* = 0.012), post-walk left knee discomfort (*p* = 0.044) and pre-leg extension left knee discomfort (*p* = 0.03). In addition, post-walk right knee discomfort approached significance (*p* = 0.09, effect size (d = 0.05)) but did not achieve a statistically significant group × time interaction effect. Furthermore, when examining within-group changes only the MP group reported any significant changes from the beginning to the end of the study protocol and in all instances, assessments of pain or discomfort decreased while indicators of health, stability or mobility improved (see Table 4).

### 3.4. Profile of Mood States

Of the 35 values recorded as part of the POMS assessment, the vigorous subset revealed a significant (*p* = 0.02, 95% CI: −0.52, 0.28) group × time interaction. Follow-up within-group paired samples *t*-tests revealed a significant reduction in the vigorous score in the MP group (Baseline: 1.63 ± 1.10 vs. Post: 1.13 ± 1.23, *p* = 0.03) while the placebo group exhibited no change (Baseline: 1.53 ± 1.05 vs. Post: 1.62 ± 0.95, *p* = 0.52). In addition, four other variables: energetic (*p* = 0.06, effect size (d = 0.58)), confused (*p* = 0.08, effect size (d = 0.46)), helpless (*p* = 0.10, effect size (d = 0.44)) and weary (*p* = 0.09, effect size (d = 0.47)) approached significance and in each instance, mean changes revealed a favorable outcome for the MP group.

### 3.5. Exercise Performance

**Figure 3:**
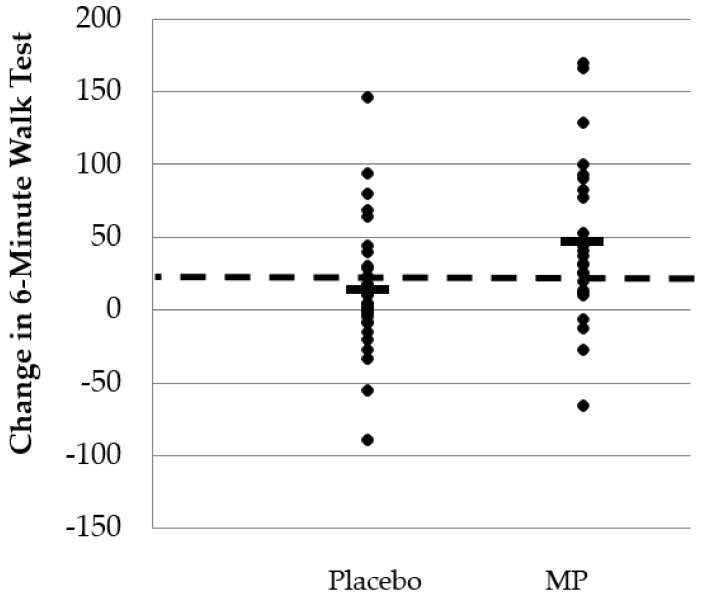
Changes in performance of 6-min walk test after eight weeks of supplementation. The placebo group (*n* = 34) reported an average increase in distance covered of 1.39 m. Of the 34 scores in the PLA group, 9 (26.5%) scores experienced a performance improvement greater than the entire study average. The proprietary milk protein concentrate group (*n* = 24) reported an average increase in distance covered of 4.74 m. Of the 24 scores in the proprietary milk protein concentrate group, 14 (58.3%) scores experienced a performance improvement greater than the entire study average. Dotted line = average change in 6-min walk distance for entire study cohort. Black horizontal bars = average change in 6-min walk distance for each study group.

Significant group × time interaction effects were found for changes in 6-min walk test performance, favoring the MP group (*p* = 0.012; 95% CI: (−60, −7.7). A significant within-group change was observed for MP (Baseline: 566 ± 111 vs. Post: 614 ± 120 m, *p* < 0.001), which equated to a 9% increase in distance walked while the PLA group experienced a 2% increase in performance (Baseline: 589 ± 121 vs. Post: 603 ± 122 m, *p* = 0.059). Changes in walk test performance are provided in Figure 3.

### 3.6. Clinical Safety and Cartilage Breakdown

No significant group × time interactions were found for any of the measured variables (Table 5). Significant within-group changes were noted for a small number of variables but in all instances, the values fell within normal clinical reference ranges. No significant group × time interaction effect (95% CI on the difference between groups: −38 to 33 ng/mL, *p* = 0.89) was identified for changes in cartilage oligomeric matrix protein (COMP). Furthermore, no within-group changes in COMP data across time were seen for PLA (Baseline: 208 ± 93 vs. Post: 206 ± 77 ng/mL, *p* = 0.70) and MP (Baseline: 206 ± 90 vs. Post: 213 ± 88 ng/mL, *p* = 0.61).

## 4. Discussion

**Figure 4:**
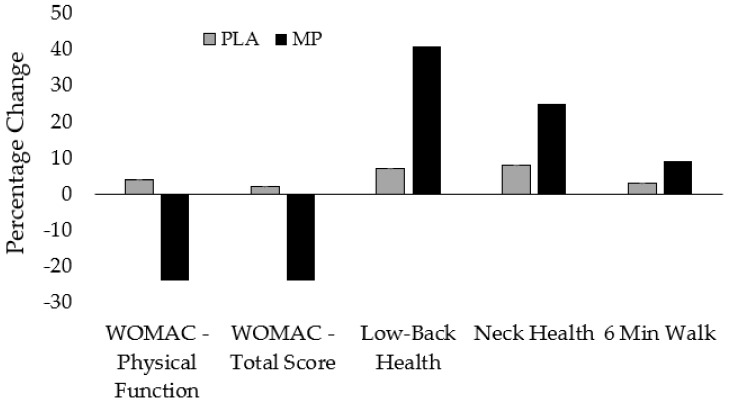
Percentage change in PLA and MP for WOMAC—Physical Function, WOMAC—Total Score, Low-Back Health, Neck Health and 6-Minute Walk Test Performance. Grey bars = PLA; Black bars = MP (proprietary milk protein concentrate).

Using a randomized, double-blind, placebo-controlled design, the purpose of the present study was to assess the impact of a proprietary milk protein concentrate from hyperimmunized cows on changes in joint health, joint pain, physical function, walking performance, quality of life and other measures of affect in non-osteoarthritic men and women who reported having mild to moderate knee pain during physical activity. The primary findings of this investigation indicate that treatment with proprietary milk protein concentrate resulted in significantly greater improvements in several components within the WOMAC assessment, walking performance, as well as self-reported levels of pain, discomfort, stiffness and stability (see Figure 4).

Previous research has established the ability of various dairy products to improve joint pain and associated symptoms in osteoarthritic and rheumatoid arthritic populations [15], improve cholesterol [9] and reduce inflammation [10,11]. While these studies are encouraging, they did not measure potential effects on functional endpoints in non-osteoarthritic individuals.

To date, only two studies have used rigorous study designs to assess the potential efficacy of concentrated milk protein produced from hyperimmunized cows. Colker et al. [16] supplemented 31 adults who were previously diagnosed with knee osteoarthritis with either a placebo or a proprietary milk protein concentrate in a randomized, double-blind manner. All participants completed similar assessments to those in the present study (VAS, WOMAC, etc.) at the beginning and after six weeks of supplementation. In agreement with our findings, significant treatment effects in favor of proprietary milk protein concentrate supplementation were found for knee pain, sport function and quality of life. An additional investigation by Zenk et al. [17] compared the impact of ingesting proprietary milk protein concentrate, glucosamine sulfate or a placebo in men and women (range: 34–86 years) previously diagnosed with osteoarthritis. As in the present study, osteoarthritis symptoms assessed via WOMAC improved in the proprietary milk protein concentrate group, with similar but less pronounced effects occurring in the glucosamine group. A key difference between the present study and these previous investigations was our use of non-osteoarthritic individuals as subjects. Indeed, improvements in joint health were noted in our non-osteoarthritic participants, along with additional noteworthy findings. First, we reported for the first time, perceived improvements in overall back and neck health as well as significant improvements in six-minute walk performance in subjects who supplemented with the proprietary milk protein concentrate. While we recognize that other forms of exercise may or may not yield similar outcomes, considering the critical role of walking performance on quality of life in present day society, the positive changes reported in the present study are quite encouraging. Finally, in agreement with previous studies, milk protein concentrate supplementation was well tolerated and all markers of safety remained within clinically accepted normative values throughout the intervention. Furthermore, no adverse events occurred throughout the study.

Considering the wide array of beneficial changes in the WOMAC and VAS data, we were somewhat surprised to find no changes in cartilage oligomeric matrix protein (COMP), a marker of cartilage breakdown. COMP is found in human chondrocytes and is known to increase after knee injury and in early stages of osteoarthritis [22,23] and COMP levels have been shown to increase in endurance-trained runners [24]. Data from the present study, however, did not uncover any significant changes in COMP from supplementation. This is likely due to the fact that our participants had not recently suffered some form of joint injury, were not diagnosed with osteoarthritis and were not currently completing high volumes of endurance training. It is possible that prescribing some form of exercise program may lead to changes in COMP levels but this remains beyond the scope of our study protocol.

A few key strengths of our study design are worth highlighting. First, the present investigation is the largest randomized, double-blind, placebo-controlled study to date on concentrated milk protein derived from milk produced in hyperimmunized cows. The previous work of Zenk and Colker used 10–15 participants per group while our study finished with 58 participants. Additionally, the participants recruited in the present study were younger and were required to have no pain at rest and only mild to moderate pain after physical exertion (i.e., had no previous medical diagnosis of arthritis). In addition, the Zenk and Colker studies reported on changes in concentrated milk protein ingestion in subjects that had already been diagnosed with osteoarthritis in at least one joint, making it tempting to speculate that a larger window of improvement existed in these previous studies as opposed to our cohort. This key point also speaks to the potential impact the present findings may have on the much larger population of people beginning to experience knee pain but not yet diagnosed with arthritis. Finally, the present study was the first to examine and report on improvements in a measure of physical performance (6-minute walk) as well as changes in a biomarker of cartilage breakdown.

We acknowledge our study has limitations. For starters, our results are specific to a middle-aged population with no resting knee pain, no previous diagnosis of joint disease and only mild to moderate joint pain with physical exertion. Thus, extrapolating our findings to other populations should be made with caution. The duration of the present study was only eight weeks and while this timeline extends previous findings, a longer study duration (16–24 weeks) is recommended in future studies. Finally, more accurate monitoring of physical activity is recommended to determine if early improvements in function and pain lead to natural increases in habitual physical activity and quality of life.

In conclusion, eight weeks of supplementing with a proprietary milk protein concentrate results in significant improvements in walking performance as well as several individual components of the WOMAC, the physical function subscale and the total WOMAC score. In addition, proprietary milk protein concentrate also improved self-reported indications of pain, discomfort, stiffness, soreness, function and quality of life.

## 5. Declarations

### 5.1. Ethics Approval

All participants read and signed an IRB-approved informed consent to participate document prior to their participation in the study (Integreview, Austin, TX, USA; approval date: December 13, 2017). NOTE: This statement is also found in the ‘Study Participants’ section of the manuscript.

### 5.2. Statement on Consent to Participate

All participants read and signed an IRB-approved informed consent to participate document prior to their participation in the study (Integreview, Austin, TX; approval date: December 13, 2017). NOTE: This statement is also found in the ‘Study Participants’ section of the manuscript.

### 5.3. Consent to Publish

This section is not applicable as our manuscript does not contain any individual or identifying data.

### 5.4. Availability of Data and Materials

The data and materials for this manuscript are not scheduled to be made publicly available due to the proprietary nature of the investigated materials. Contractually, the data is owned by Stolle Milk Biologics, Inc.

### 5.5. Competing Interests

Ziegenfuss has no conflict in terms of financial or business interests related to this product. Ziegenfuss has received grants and contracts to conduct research on dietary supplements; has served as a paid consultant for industry; has received honoraria for speaking at conferences and writing lay articles about protein and other sports nutrition ingredients (but not the product investigated in this study); receives royalties from the sale of several sports nutrition products (none related to the product examined in the present study); and has served as an expert witness on behalf of the plaintiff and defense in cases involving dietary supplements. Ziegenfuss is also co-inventor on multiple patent applications within the field of dietary supplements, applied nutrition and bioactive compounds. Lopez has no conflict in terms of financial or business interests related to this product. Lopez is an officer and member of The Center for Applied Health Sciences, a privately held contract research organization that has received external funding from companies that does business in the dietary supplement, natural products, medical foods and functional foods and beverages industry. He is the co-founder and member of Supplement Safety Solutions, LLC., serving as an independent consultant for regulatory compliance, safety surveillance and Nutravigilance to companies in the dietary supplement and functional foods industry but not the sponsor of the current research. Lopez is also co-inventor on multiple patent applications within the field of dietary supplements, applied nutrition and bioactive compounds. Kerksick has no conflict in terms of financial or business interests related to this product. Kerksick has received external grant funding from companies that do business in the nutrition and sports nutrition sectors. He has received compensation to speak and prepare scientific manuscripts including white papers and marketing copy on topics related to sports nutrition. He has and continues to serve in advisory roles to various sport nutrition and nutrition companies. Kedia, Sandrock and Raub all report no conflicts of interest.

## Figures and Tables

**Table 1:** Overview of Research Design.

Procedure	Visit 1 Screening	Visit 2 (Week 0)	Visit 3 (Week 4)	Visit 4 (Week 8)
Informed Consent	X			
Inclusion/Exclusion Criteria	X			
Medical History	X			
Physical Exam	X			
Height and weight	X			
Vitals (HR, BP)	X			
Blood Analyses (CBC, CMP w/lipids)	X			X
COMP (Cartilage breakdown biomarker)		X	X	X
WOMAC scales		X	X	X
3 Day Diet Records		X	X	X
Profile of Mood States		X	X	X
Visual Analog Scales		X	X	X
Physical Activity	X	X	X	X
Functional Capacity Test	X		X	X
Protocol Compliance (pill counts)			X	X
Adverse Events Monitoring		X	X	X

X = procedure performed; BP = Blood pressure; HR = heart rate; CMP = Comprehensive metabolic panel; CBC = Complete blood counts; COMP = Cartilage oligometric matrix protein.

**Table 2:** Baseline Demographics, WOMAC Scores and Dietary Data.

		MP (*n* = 24)	PLA (*n* = 34)	Sig.
Age (years)				
	Males	41.1 ± 10.0 (*n* = 11)	43.3 ± 8.4 (*n* = 19)	0.54
	Females	48.5 ± 7.9 (*n* = 13)	44.3 ± 10.7 (*n* = 15)	
Height (centimeters)				
	Males	179.4 ± 3.1	175.2 ± 7.8	0.42
	Females	164.7 ± 7.7	161.7 ± 8.6	
Body Mass (kilograms)				
	Males	93.9 ± 12.1	87.6 ± 10.8	0.59
	Females	73.1 ± 12.4	71.4 ± 14.0	
Body Mass Index (kg/m^2^)				
	Males	29.1 ± 3.5	28.6 ± 3.5	0.89
	Females	27.2 ± 5.3	27.5 ± 6.3	
Systolic Blood Pressure (mm Hg)				
	Males	123.3 ± 10.1	126.9 ± 8.8	0.29
	Females	113.8 ± 12.8	114.8 ± 12.1	
Diastolic Blood Pressure (mm Hg)				
	Males	79.9 ± 6.6	77.3 ± 8.1	0.57
	Females	73.3 ± 9.3	72.0 ± 9.4	
Resting HR (beats/min)				
	Males	69.6 ± 14.3	66.9 ± 11.0	0.70
	Females	65.9 ± 8.8	65.8 ± 8.2	
6-Minute Walk Test (meters)				
	Males	587.9 ± 146.2	623.3 ± 133.7	0.47
	Females	547.5 ± 69.6	544.4 ± 87.3	
WOMAC Total Stiffness				
	Males	2.91 ± 1.22	3.21 ± 1.23	0.90
	Females	3.39 ± 1.04	3.20 ± 1.15	
WOMAC Physical Function				
	Males	21.6 ± 11.4	23.5 ± 10.0	0.70
	Females	18.2 ± 9.2	17.2 ± 7.1	
WOMAC Overall Total				
	Males	30.6 ± 15.1	33.4 ± 13.6	0.71
	Females	26.0 ± 9.9	27.3 ± 11.8	

MP = proprietary milk protein concentrate; PLA = placebo. Sig. = *p*-value for between-group difference using independent *t*-test with genders collapsed within each group. Mm Hg = millimeters of mercury; kg = kilograms; m^2^ = meters squared; WOMAC = Western Ontario & McMaster Universities Osteoarthritis Index.

**Table 3:** WOMAC Data.

Variables	*n*	Baseline	Post	Within *p* Value	Between-Group Comparison		
					Mean Difference ^†^	95% CI	*p* Value
*WOMAC Stiffness—In Morning*							
PLA MP	34 24	1.50 ± 0.66 1.54 ± 0.78	1.35 ± 0.81 1.08 ± 0.50	0.30 0.005	0.311	(−0.11, 0.73)	0.14
*WOMAC Stiffness—After Being Immobile*							
PLA MP	34 24	1.71 ± 0.68 1.63 ± 0.65	1.50 ± 0.96 1.29 ± 0.81	0.21 0.043	0.127	(−0.34, 0.59)	0.59
*WOMAC Total Stiffness Score*							
PLA MP	34 24	3.21 ± 1.18 3.17 ± 1.13	2.85 ± 1.62 2.38 ± 1.10	0.20 0.004	0.439	(−0.32, 1.20)	0.25
*WOMAC Discomfort—Walking on Flat Surface*							
PLA MP	34 24	1.12 ± 0.64 0.96 ± 0.75	1.12 ± 0.81 0.63 ± 0.65	0.99 0.043	0.333	(−0.10, 0.77)	0.13
*WOMAC Discomfort—Going Up or Down Stairs*							
PLA MP	34 24	2.00 ± 0.65 1.96 ± 0.81	1.82 ± 1.14 1.50 ± 0.83	0.42 0.02	0.281	(−0.33, 0.89)	0.36
*WOMAC Discomfort—At Night While in Bed*							
PLA MP	34 24	0.91 ± 0.71 1.04 ± 0.91	0.97 ± 0.90 0.71 ± 0.69	0.64 0.04	0.392	(−0.007, 0.79)	0.054
*WOMAC Discomfort—Sitting or Lying*							
PLA MP	34 24	1.00 ± 0.78 0.88 ± 0.80	0.94 ± 0.89 0.71 ± 0.69	0.69 0.30	0.108	(−0.33, 0.54)	0.62
*WOMAC Discomfort—Standing Upright*							
PLA MP	34 24	1.15 ± 0.86 1.08 ± 0.50	1.12 ± 0.88 0.71 ± 0.69	0.85 0.004	0.346	(−0.07, 0.77)	0.11
*WOMAC Total Discomfort Score*							
PLA MP	34 24	6.18 ± 2.95 5.92 ± 2.75	5.97 ± 4.20 4.25 ± 2.58	0.76 0.006	1.46	(−0.40, 3.32)	0.12
*WOMAC Physical Function—Descending Stairs*							
PLA MP	34 24	1.71 ± 0.63 1.71 ± 1.04	1.79 ± 1.20 1.17 ± 0.92	0.65 0.004	0.630	(0.09, 1.17)	0.02 *
*WOMAC Physical Function—Ascending Stairs*							
PLA MP	34 24	1.68 ± 0.77 1.75 ± 0.79	1.68 ± 1.15 1.21 ± 0.88	0.99 0.02	0.54	(−0.03, 1.11)	0.062
*WOMAC Physical Function—Rising to Sitting*							
PLA MP	34 24	1.35 ± 0.73 1.39 ± 0.72	1.41 ± 0.82 1.00 ± 0.74	0.70 0.08	0.45	(−0.06, 0.96)	0.08
*WOMAC Physical Function—Standing*							
PLA MP	34 24	0.97 ± 0.81 0.96 ± 0.69	1.00 ± 0.90 0.58 ± 0.58	0.79 0.02	0.41	(0.05, 0.76)	0.03 *
*WOMAC Physical Function—Bending to Floor*							
PLA MP	34 24	1.44 ± 0.66 1.17 ± 0.82	1.35 ± 0.92 0.75 ± 0.74	0.61 0.02	0.33	(−0.16, 0.82)	0.18
*WOMAC Physical Function—Walking on Flat Surface*							
PLA MP	34 24	1.00 ± 0.82 0.92 ± 0.78	0.94 ± 0.89 0.46 ± 0.59	0.71 0.008	0.40	(−0.06, 0.86)	0.09
*WOMAC Physical Function—Getting in Car*							
PLA MP	34 24	1.09 ± 0.75 1.13 ± 0.85	1.24 ± 0.96 0.63 ± 0.71	0.28 0.001	0.65	(0.25, 1.04)	0.002 *
*WOMAC Physical Function—Shopping*							
PLA MP	34 24	0.97 ± 0.76 0.87 ± 0.87	1.03 ± 0.90 0.35 ± 0.49	0.62 0.002	0.58	(0.20, 0.96)	0.004 *
*WOMAC Physical Function—Putting on Socks*							
PLA MP	34 24	0.88 ± 0.81 0.92 ± 0.83	1.03 ± 0.87 0.42 ± 0.65	0.23 0.005	0.65	(0.25, 1.04)	0.002 *
*WOMAC Physical Function—Rising from Bed*							
PLA MP	34 24	1.09 ± 0.71 1.13 ± 0.68	1.12 ± 0.88 0.83 ± 0.76	0.83 0.13	0.32	(−0.13, 0.77)	0.16
*WOMAC Physical Function—Removing Socks*							
PLA MP	34 24	0.94 ± 0.78 0.86 ± 0.83	1.06 ± 0.92 0.36 ± 0.58	0.33 0.005	0.62	(0.23, 1.00)	0.002 *
*WOMAC Physical Function—Moving in Bed*							
PLA MP	34 24	0.85 ± 0.74 1.04 ± 0.95	0.91 ± 0.90 0.54 ± 0.72	0.60 0.001	0.56	(0.21, 0.91)	0.002 *
*WOMAC Physical Function—Getting In and Out of Bath*							
PLA MP	34 24	0.97 ± 0.76 0.78 ± 0.80	1.00 ± 0.85 0.35 ± 0.57	0.77 0.002	0.46	(0.15, 0.78)	0.005 *
*WOMAC Physical Function—Sitting*							
PLA MP	34 24	0.88 ± 0.73 0.83 ± 0.82	0.97 ± 0.90 0.58 ± 0.72	0.48 0.08	0.34	(−0.03, 0.71)	0.074
*WOMAC Physical Function—Getting On and Off of Toilet*							
PLA MP	34 24	1.00 ± 0.75 0.83 ± 0.82	1.12 ± 0.96 0.50 ± 0.72	0.33 0.008	0.45	(0.11, 0.80)	0.011 *
*WOMAC Physical Function—Heavy Domestic Tasks*							
PLA MP	34 24	2.15 ± 0.70 1.96 ± 0.75	1.97 ± 1.09 1.50 ± 0.72	0.33 0.02	0.28	(−0.24, 0.80)	0.28
*WOMAC Physical Function—Light Domestic Tasks*							
PLA MP	34 24	1.79 ± 0.70 1.50 ± 0.66	1.52 ± 1.09 0.79 ± 0.78	0.20 <0.001	0.44	(−0.12, 0.99)	0.12
*WOMAC Physical Function Total Score*							
PLA MP	34 24	20.7 ± 9.3 19.8 ± 10.2	21.0 ± 13.8 12.0 ± 8.6	0.87 <0.001	8.0	(2.8, 13.3)	0.004 *
*WOMAC Total Score*							
PLA MP	34 24	30.1 ± 12.5 28.8 ± 13.2	29.9 ± 19.4 18.6 ± 11.7	0.92 <0.001	9.94	(2.5, 17.4)	0.01 *

PLA = Placebo; MP = proprietary milk protein concentrate. 95% CI = 95% confidence interval of the difference between MP and PLA. **^†^** Absolute difference in delta values between groups. Within *p*-value derived from factorial ANOVA with repeated measures on time. Between-group *p*-values were calculated using independent *t*-tests using the delta value (week 8–week 0). * indicates statistical significance.

**Table 4:** Visual Analog Scales to Assess Pain and Discomfort.

Variables	*n*	Baseline	Post	Within *p* Value	Between−Group Comparison		
					Mean Difference ^†^	95% CI	*p* Value
*Discomfort in Right Knee in Past 1–3 Weeks*							
PLA MP	34 24	7.4 ± 17.5 4.1 ± 2.9	3.6 ± 3.6 3.1 ± 2.7	0.23 0.04	−2.68	(−10.1, 4.8)	0.47
*Discomfort in Left Knee in Past 1–3 Weeks*							
PLA MP	34 24	3.2 ± 2.4 4.4 ± 2.5	2.3 ± 2.4 3.2 ± 2.7	0.025 0.01	0.27	(−0.92, 1.45)	0.65
*Knee Mobility*							
PLA MP	34 24	6.6 ± 1.9 6.8 ± 2.2	6.6 ± 2.1 7.6 ± 1.5	0.89 0.12	−0.92	(−2.09, 0.25)	0.12
*Knee Stability*							
PLA MP	34 24	6.5 ± 1.8 6.3 ± 2.0	6.5 ± 1.2 8.2 ± 1.0	0.89 <0.001	−1.85	(−2.82, −0.88)	0.001 *
*Whole-Body Joint Health*							
PLA MP	34 24	6.4 ± 1.8 6.6 ± 1.9	6.9 ± 1.9 7.9 ± 1.1	0.21 0.007	−0.86	(−1.95, 0.23)	0.12
*Low-Back Health*							
PLA MP	34 24	6.6 ± 2.2 6.1 ± 1.9	6.3 ± 1.7 7.6 ± 1.6	0.35 0.009	−1.78	(−2.90, −0.66)	0.002 *
*Neck Health*							
PLA MP	34 24	6.7 ± 1.8 6.3 ± 2.4	6.8 ± 1.8 8.0 ± 1.3	0.75 0.006	−1.53	(−2.71, −0.36)	0.012 *
*Level of Right Knee Discomfort: Pre-Walk*							
PLA MP	34 24	3.4 ± 3.1 2.8 ± 2.7	3.6 ± 3.5 2.8 ± 2.5	0.33 0.99	0.28	(−0.70, 1.27)	0.57
*Level of Left Knee Discomfort: Pre-Walk*							
PLA MP	34 24	2.1 ± 1.9 3.2 ± 2.6	2.2 ± 2.3 2.5 ± 2.4	0.87 0.10	0.75	(−0.22, 1.73)	0.13
*Level of Right Knee Discomfort: Post-Walk*							
PLA MP	34 24	3.8 ± 3.2 3.1 ± 3.0	4.0 ± 3.6 2.5 ± 2.4	0.58 0.11	0.76	(−0.13, 1.64)	0.09
*Level of Left Knee Discomfort: Post-Walk*							
PLA MP	34 24	2.5 ± 2.1 3.6 ± 2.5	2.3 ± 2.6 2.5 ± 2.4	0.58 0.003	0.97	(0.03, 1.91)	0.044 *
*Level of Right Knee Discomfort: Pre Leg Extension*							
PLA MP	34 24	3.8 ± 3.2 3.1 ± 3.0	4.0 ± 3.6 2.6 ± 2.5	0.45 0.19	0.74	(−0.17, 1.65)	0.11
*Level of Left Knee Discomfort: Pre-Leg Extension*							
PLA MP	34 24	2.4 ± 2.2 3.6 ± 2.7	2.3 ± 2.4 2.4 ± 2.3	0.80 0.003	1.11	(0.14, 2.08)	0.03 *
*Level of Right Knee Discomfort: Post Leg Extension*							
PLA MP	34 24	4.5 ± 3.4 4.2 ± 3.2	4.1 ± 3.7 3.5 ± 3.0	0.21 0.21	0.24	(−0.95, 1.42)	0.69
*Level of Left Knee Discomfort: Post Leg Extension*							
PLA MP	34 24	3.1 ± 2.6 4.7 ± 2.7	2.5 ± 2.4 3.4 ± 2.7	0.09 0.01	0.57	(−0.38, 1.91)	0.18
*Framingham Score*							
PLA MP	34 24	38.6 ± 8.9 37.7 ± 9.2	38.9 ± 9.7 38.0 ± 10.0	0.59 0.69	−0.04	(−1.83, 1.75)	0.97

PLA = Placebo; MP = proprietary milk protein concentrate. 95% CI = 95% confidence interval of the difference between MP and PLA. ^†^ Absolute difference in delta values between groups. Within p-value derived from factorial ANOVA with repeated measures on time. Between-group p-values were calculated using independent t-tests using the delta value (week 8–week 0). * indicates statistical significance.

**Table 5:** Serum and Whole Blood Clinical Markers of Safety.

Variables	*n*	Baseline (Week 0)	Post (Week 8)	Within *p* Value	Between-Group Comparison		
					Mean Difference	95% CI	*p* Value
*White Blood Cell Count (cells/L)*							
PLA MP	34 24	5.54 ± 1.41 5.85 ± 1.43	5.20 ± 1.48 5.65 ± 1.24	0.045 0.56	−0.14 ± 0.37	(−0.90, 0.61)	0.71
*Red Blood Cell Count (cells/L)*							
PLA MP	34 24	4.89 ± 0.43 4.69 ± 0.42	4.91 ± 0.51 4.71 ± 0.35	0.57 0.72	0.003 ± 0.06	(−0.12, 0.12)	0.95
*Hemoglobin (g/dL)*							
PLA MP	34 24	14.7 ± 1.4 14.3 ± 1.6	14.5 ± 1.4 14.1 ± 1.3	0.23 0.11	2.86 ± 1.98	(−1.12, 6.83)	0.16
*Hematocrit (%)*							
PLA MP	34 24	41.6 ± 3.0 40.7 ± 4.4	42.3 ± 3.6 41.2 ± 3.4	0.03 0.21	0.06 ± 0.18	(−0.30, 0.41)	0.75
*Glucose (mg/dL)*							
PLA MP	34 24	86.4 ± 7.0 91.3 ± 7.4	89.7 ± 10.9 95.2 ± 8.9	0.07 0.01	−0.51 ± 2.3	(−5.02, 4.0)	0.82
*Blood Urea Nitrogen (BUN) (mg/dL)*							
PLA MP	34 24	14.5 ± 4.3 15.7 ± 4.7	14.9 ± 4.0 15.3 ± 4.0	0.66 0.63	0.70 ± 1.09	(−1.48, 2.88)	0.52
*Creatinine (mg/dL)*							
PLA MP	34 24	0.87 ± 0.14 0.88 ± 0.17	0.91 ± 0.14 0.88 ± 0.17	0.04 0.71	0.03 ± 0.02	(−0.02, 0.08)	0.23
*BUN: Creatinine Ratio*							
PLA MP	34 24	16.8 ± 4.6 17.9 ± 4.9	16.3 ± 3.3 17.5 ± 4.5	0.58 0.71	−0.096 ± 1.30	(−2.70, 2.51)	0.94
*Sodium (mEq/L)*							
PLA MP	34 24	141.7 ± 2.0 141.3 ± 2.3	140.3 ± 2.6 140.2 ± 1.9	0.009 0.012	−0.33 ± 0.69	(−1.71, 1.05)	0.64
*Potassium (mEq/L)*							
PLA MP	34 24	4.4 ± 0.25 4.3 ± 0.26	4.4 ± 0.27 4.3 ± 0.23	0.62 0.72	−0.014 ± 0.07	(−0.16, 0.13)	0.84
*Chloride (mEq/L)*							
PLA MP	34 24	102.5 ± 2.2 101.9 ± 2.2	102.6 ± 3.0 101.6 ± 1.8	0.87 0.54	0.42 ± 0.79	(−1.16, 2.00)	0.59
*Carbon Dioxide (mEq/L)*							
PLA MP	34 24	24.1 ± 1.8 23.5 ± 2.1	23.8 ± 2.3 23.9 ± 2.3	0.48 0.28	−0.72 ± 0.56	(−1.84, 0.40)	0.20
*Calcium (mg/dL)*							
PLA MP	34 24	9.26 ± 0.23 9.28 ± 0.33	9.27 ± 0.27 9.26 ± 0.34	0.92 0.78	0.02 ± 0.08	(−0.15, 0.19)	0.79
*Protein (g/dL)*							
PLA MP	34 24	6.99 ± 0.42 9.81 ± 0.43	6.92 ± 0.37 6.94 ± 0.44	0.30 0.74	−0.09 ± 0.1	(−0.29, 0.10)	0.35
*Albumin (g/dL)*							
PLA MP	34 24	4.47 ± 0.28 4.53 ± 0.33	4.48 ± 0.18 4.52 ± 0.28	0.79 0.95	0.16 ± 0.08	(−0.14, 0.17)	0.83
*Globulin (g/dL)*							
PLA MP	34 24	2.52 ± 0.32 2.39 ± 0.23	2.44 ± 0.35 2.42 ± 0.32	0.09 0.58	−0.11 ± 0.07	(−0.24, 0.03)	0.13
*Albumin: Globulin Ratio*							
PLA MP	34 24	1.81 ± 0.24 1.92 ± 0.21	1.88 ± 0.28 1.91 ± 0.30	0.12 0.89	0.07 ± 0.06	(−0.06, 0.20)	0.30
*Bilirubin (mg/dL)*							
PLA MP	34 24	0.50 ± 0.20 0.60 ± 0.34	0.44 ± 0.20 0.48 ± 0.25	0.03 0.03	0.07 ± 0.06	(−0.05, 0.18)	0.25
*Alkaline Phosphatase (IU/L)*							
PLA MP	34 24	70.7 ± 17.3 65.5 ± 18.7	72.3 ± 18.2 67.2 ± 20.6	0.21 0.23	−0.04 ± 1.84	(−3.7, 3.6)	0.98
*Aspartate Aminotransferase (U/L)*							
PLA MP	34 24	22.4 ± 8.7 19.7 ± 4.7	23.7 ± 14.7 20.8 ± 8.2	0.83 0.56	−0.13 ± 1.23	(−2.6, 2.3)	−0.92
*Alanine Aminotransferase (U/L)*							
PLA MP	34 24	23.7 ± 14.7 20.8 ± 8.2	20.0 ± 8.9 19.8 ± 7.7	0.02 0.17	−2.65 ± 1.84	(−6.3, 1.0)	0.16
*Total Cholesterol (mg/dL)*							
PLA MP	34 24	178 ± 31 196 ± 34	174 ± 31 194 ± 40	0.21 0.72	−2.2 ± 5.4	(−13.0, 8.6)	0.68
*Triglycerides (mg/dL)*							
PLA MP	34 24	110 ± 81 119 ± 83	90 ± 74 118 ± 81	0.002 0.91	−18.4 ± 15.0	(−48.5, 11.6)	0.23
*HDL Cholesterol (mg/dL)*							
PLA MP	34 24	58 ± 19 59 ± 15	57 ± 20 56 ± 18	0.79 0.04	2.86 ± 2.0	(−1.10, 6.81)	0.15
*VLDL Cholesterol (mg/dL)*							
PLA MP	34 24	19.8 ± 10.6 23.9 ± 16.7	16.1 ± 9.1 23.5 ± 16.2	0.003 0.92	−3.48 ± 3.0	(−9.6, 2.6)	0.26
*LDL Cholesterol (mg/dL)*							
PLA MP	34 24	95 ± 30 113 ± 23	99 ± 27 115 ± 28	0.37 0.62	1.50 ± 5.4	(−9.3, 12.3)	0.78

PLA = Placebo; MP = proprietary milk protein concentrate. L = Liters; g = grams; dL = deciliter; mg = milligrams; mEq = milliequivalents; IU = International Units; U = Units of catalyzed enzyme; HDL = High-density lipoprotein; VLDL = Very low-density lipoprotein; LDL = Low-density lipoprotein; 95% CI = 95% confidence interval of the difference between MP and PLA. Within p-value derived from factorial ANOVA with repeated measures on time. Between-group p-values were calculated using independent t-tests using the delta value (week 8–week 0).
